# Solution Structures of Two Homologous Venom Peptides from *Sicarius dolichocephalus*

**DOI:** 10.1371/journal.pone.0054401

**Published:** 2013-01-14

**Authors:** Nikolaus M. Loening, Zachary N. Wilson, Pamela A. Zobel-Thropp, Greta J. Binford

**Affiliations:** 1 Chemistry Department, Lewis & Clark College, Portland, Oregon, United States of America; 2 Biology Department, Lewis & Clark College, Portland, Oregon, United States of America; Weizmann Institute of Science, Israel

## Abstract

We present solution-state NMR structures for two putative venom peptides from *Sicarius dolichocephalus*. These peptides were identified from cDNA libraries created from venom gland mRNA and then recombinantly expressed. They are the first structures from any species of *Sicarius* spiders, and the first peptide structures for any haplogyne spiders. These peptides are homologous to one another, and while they have at most only 20% sequence identity with known venom peptides their structures follow the inhibitor cystine knot motif that has been found in a broad range of venom peptides.

## Introduction

Each species of venomous spider produces a venom mixture that typically contains hundreds of peptides and proteins [1], many of which affect neuronal ion channels and cell receptors with exquisite specificity. As there are over 43,000 documented species of spiders [2], there may be more than a million pharmacologically active peptides to be discovered in spider venoms. Spider venoms have not been as extensively studied as venoms from cone snails, snakes, and scorpions even though they are predicted to have the largest wealth of chemical diversity of these lineages [3]. Amid this wealth of compounds, many of the medium-sized (35–45 residue) venom peptides share a common structural motif known as the inhibitor cystine knot (ICK), which usually incorporates three or four disulfide bonds along with two or three anti-parallel **β** sheets [4], [5]. The conserved cysteines of this motif result in a stable scaffold upon which a great deal of peptide diversity has evolved by hypermutation of the intercysteine regions [6], [7]. As a consequence of this diversity, venom peptides vary considerably in target and species-specificity.

In this paper, we present solution-state nuclear magnetic resonance (NMR) structures two peptides identified from the spider *Sicarius dolichocephalus*, U_1_-sicaritoxin-Sd1a and U_2_-sicaritoxin-Sd1a. For brevity we refer to these as S64 and S67 throughout this manuscript. These peptides were identified from cDNA libraries that were created from venom gland mRNA. Based on the conserved pattern of six cysteines in these peptides, their origin, and their structures following the ICK motif, we believe that these peptides are likely to be neurotoxins, in which case it is probable that they target neuronal ion channels. *S. dolichocephalus* is one of 22 *Sicarius* spider species that, along with approximately 100 *Loxosceles* species, make up the sicariid family. The sicariid family is over 100 million years old [8] and is part of the Haplogynae series of araneomorph spiders [9]. They are ground dwelling generalist foragers that bury themselves in sand in dry regions of southern Africa and South/Central America. Our structures for S64 and S67 are the first protein or peptide structures from *Sicarius* spiders, and join the crystal structure of the protein sphingomyelinase D from *Loxosceles laeta* [10] as the only structures to date from the sicariid family and from any haplogyne spider. Therefore, characteristics of these peptides and their similarity to others provide key insight into the phylogenetic conservation of venom peptides in spiders.

## Methods

### Library Construction

*S. dolichocephalus* spiders were collected in Ruacana Falls, Namibia, Africa by GJB and colleagues; details of specific collecting localities and voucher specimens are available from GJB upon request. All necessary permits were obtained for the described field studies. Permits were obtained from Namibia's Ministry of Environment and Tourism (collecting permit number 945/2005, export permit number 54259). If animals were not mature when collected, we reared them to maturity in the lab at 35% humidity and 24°C. We extracted venom from spiders using electrical stimulation [11]. After three days, which is a sufficient amount of time for the venom glands to refresh their supply, we anesthetized the spiders using CO_2_, pulled the venom glands, and flash-froze them immediately in liquid nitrogen.

We isolated total RNA from a single *S. dolichocephalus* spider using the ChargeSwitch® Total RNA Cell Kit (Invitrogen). Using this RNA, we constructed a cDNA library using the SMART^™^ cDNA library construction kit (Clontech), which is optimized for making complete, full-length cDNAs from small amounts of starting material. We used 72 ng of total RNA from *S. dolichocephalus* for first strand synthesis of cDNA and followed the manufacturer's protocol for library construction, with the exception of using CHROMA SPIN^™^ columns (Clontech) for size fractionation and purification of cDNAs. To capture small toxins and peptides in addition to proteins we used the NucleoTraP®CR kit (Macherey-Nagel), which binds and purifies all DNA fragments larger than 100 bp to a silica matrix. Upon ligation into the λTriplEx2 vector, we packaged the cDNAs into MaxPlax^™^ lambda packaging extracts (Epicentre) according to the manufacturer's protocol and infected *Escherichia coli* XL1-Blue bacterial cells (Clontech). We eluted phage particles from plaques by poking a pipet tip through each plaque and stirring it into 500 μL of a buffered solution (100 mM NaCl/10 mM MgSO_4_/50 mM Tris-Cl, pH 7.5/0.01% gelatin) with 20 µL of chloroform. These samples were incubated for 2 hrs at room temperature and stored at 4°C.

We identified cDNA inserts larger than 500 bp with a PCR screen that used λTriplEx2 primers that flank the insert (Clontech, 0.2 µM final concentration), 2X MasterAmp^™^ F PCR Premix (Epicentre, 1× final concentration) and *Taq* polymerase (New England Biolabs, 0.05 U/µL final concentration). We performed PCR with an initial denaturing step of 95°C for 1.5 min followed by 30 cycles of 95°C for 1 min/66°C for 1 min/72°C for 1 min, and then a final extension at 72°C for 7 min. For sequencing we converted the λ vector-containing products to plasmid products (pTriplEx2) using *E. coli* BM25.8 cells provided with the library kit (Clontech). Plasmids were sequenced with the 5′- and 3′-sequencing primers specified by the TriplEx2 vector map on an Applied Biosystems 3730xl DNA Analyzer at the Genomic Analysis and Technology Core (University of Arizona).

### Sequence analysis

We trimmed vector sequence and assembled all sequences using Sequencher (version 4.7, Gene Codes Corp.). We identified S64 and S67 as potential toxins by searching for homologs in the ArachnoServer 2.0 spider toxin database [12] (http://www.arachnoserver.org) using tBLASTx [13]. We also searched the NCBI nr and expressed tag (EST) databases (http://blast.ncbi.nlm.nih.gov/) to see if we could find more similar sequences than our top hits in ArachnoServer. We aligned amino acid sequences with one another and other toxin sequences identified in homology searches using ClustalX [14] and refined the alignment manually in MacClade [15] (version 4.08) using conserved cysteines to anchor the alignment. We predicted the signal peptide sequence using SignalP 4.0 [16]. To identify the mature peptide sequence we primarily relied on alignment with known spider venoms to determine the cleavage site. The sequences for S64 and S67 were added to the Arachnoserver database, where they have the names U_1_-sicaritoxin-Sd1a and U_2_-sicaritoxin-Sd1a following the database's unified nomenclature [17].

### Peptide Expression and Purification

**Figure 1:**
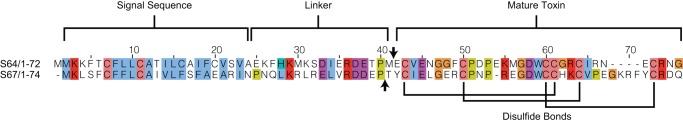
Sequence alignment for the translated sequences of S64 and S67. This sequence alignment for S64 and S67 illustrates the signal sequence, linker, and mature toxin. Small arrows show the predicted cleavage sites for the mature toxins. The experimentally determined disulfide bond connectivity shown applies to both peptides. Sequences were aligned using ClustalX 2.1 and visualized using JalView 2.7 [36]. The coloring makes use of the default ClustalX color scheme, which is a function of sequence identity and amino acid type.

The sequences for the mature venom peptides (using the predicted cleavage sites shown in Figure 1) were codon-optimized for expression in *E. coli*, and then synthesized and inserted into the pLICC vector by GeneArt (Invitrogen). The pLICC system (Dr. Glenn King, U. of Queensland) utilizes a pET-derived vector backbone. The expression product from the system is a fusion protein with a periplasmic tag, polyhistidine tag, and maltose binding domain tag at the N-terminus of the peptide. In addition, we engineered a TEV cleavage site immediately before the venom peptide sequence so that the peptide can be efficiently and specifically cleaved from the fusion protein. Due to the requirements for the TEV cleavage site, both toxin peptides have an additional amino acid at their N-termini relative to the mature wild-type venom peptide sequences. In the case of S64 this was a serine residue, whereas for S67 the additional residue was a glycine.

**Figure 2:**
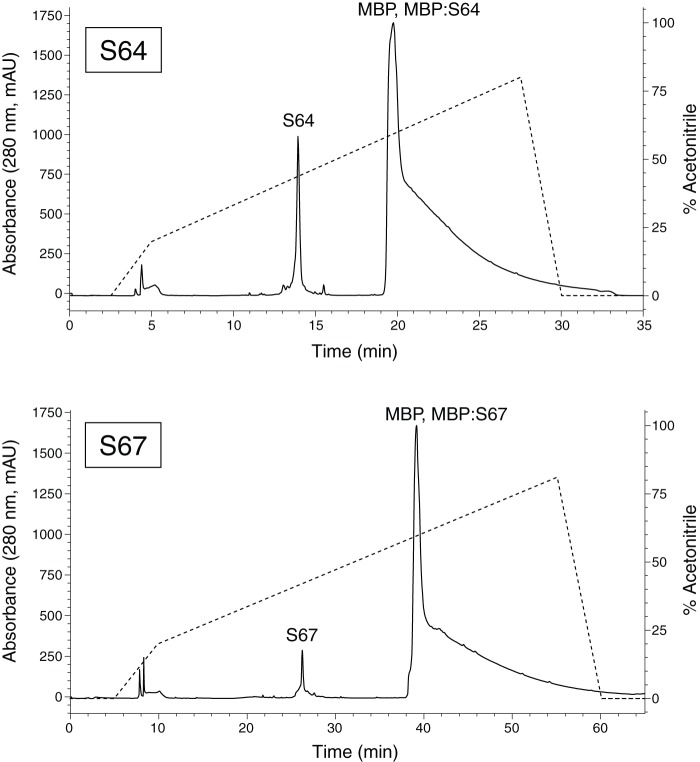
HPLC chromatograms for the cleavage reaction products from S64 (**top**) **and S67** (**bottom**)**.** The solid line corresponds to the left axis and represents the absorbance at 280 nm; the dashed line corresponds to the right axis and represents the solvent composition. In both chromatograms, the cleaved peptide is well-resolved from other peaks. The large peak that appears at high acetonitrile concentrations corresponds to a combination of the maltose binding domain (MBP) tag, uncleaved fusion protein (MBP:S64/MBP:S67) and TEV protease. Peak identities were confirmed by performing gel electrophoresis and mass spectrometry for select fractions. The flow rate was 4 mL/min (top) and 2 mL/min (bottom).

**Figure 3:**
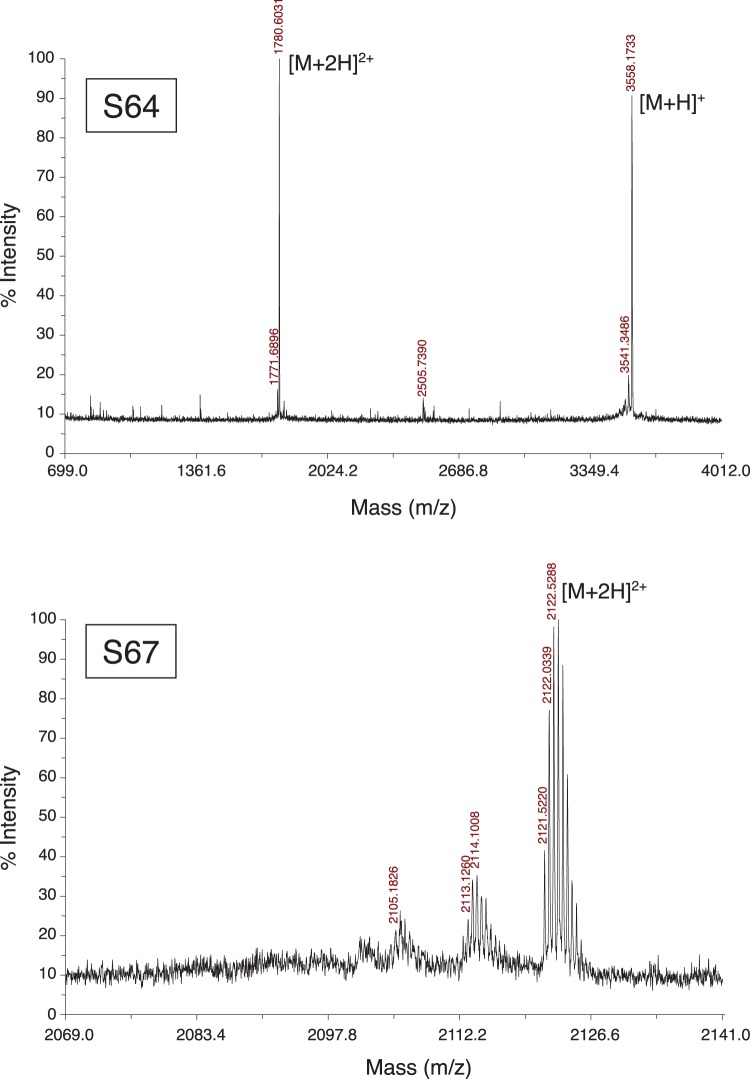
MALDI-TOF mass spectra for unlabeled S64 (**left**) **and S67** (**right**)**.** The predicted monoisotopic masses for fully oxidized S64 are 3558.3 Da for the [M+H]^+^ ion and 1779.69 Da for the [M+2H]^2+^ ion. Likewise, the predicted mass for fully oxidized S67 is 2121.41 Da for the [M+2H]^2+^ ion. The appearance of peaks at these masses indicates that the cysteines are oxidized for both S64 and S67. The additional peaks seen to the left of the [M+2H]^2+^ peak in the S67 mass spectrum correspond to ions that have dehydrated. These peaks are also present in the S64 spectrum but are difficult to discern due to the much wider scale used for this spectrum.

We used the expression vectors to transform *E. coli* BL21 (DE3) cells. Expression trials were typically performed at 22°C on a 0.5 L scale using an autoinducing minimal media [18]. To generate isotopically-labeled samples for NMR spectroscopy, we used ^15^NH_4_Cl, ^13^C_3_-glycerol, and ^13^C_6_-glucose as the primary nitrogen and carbon sources. After three days of incubation we harvested the cells by centrifugation and resuspended the pellet in BugBuster^™^ (Novagen) to lyse the cells. After ultracentrifugation of the lysate, we purified the supernatant using His GraviTrap columns (GE Healthcare) following the manufacturer's recommended procedure. Separate experiments with alternate lysis procedures (lysozyme or mechanical disruption) indicated that the fusion protein was present in the soluble fraction after ultracentrifugation. After eluting the protein from the His GraviTrap column and buffer exchange using an Amicon centrifugal filter unit (Millipore), we cleaved the purified fusion protein with TEV protease in a redox buffer of 2 mM reduced glutathione/0.2 mM oxidized glutathione. The cleavage reaction was performed for three hours at room temperature or overnight at 4°C. We then purified the products of the cleavage reaction using reverse-phase liquid chromatography (Figure 2) with a water/acetonitrile gradient and a PolyEncap A300 column (Bischoff Chromatograph). The fraction that eluted at 35% acetonitrile contained the purified venom peptide, as confirmed by MALDI-TOF mass spectrometry (Figure 3) using an Applied Biosystems 4700 Proteomics Analyzer and molecular weights predicted by the ExPASy compute pI/Mw tool (http://web.expasy.org/compute_pi/). We then lyophilized this fraction and rehydrated it in a buffer appropriate for NMR spectroscopy (95% H_2_O/5% D_2_O/20 mM sodium phosphate). The sodium phosphate buffer had a pH of 7.4 for the S64 sample and 6.0 for the S67 sample. Typical yields of fusion protein were 20–50 mg for a 0.5 L culture, resulting in 0.5–2 mg of venom peptide after the final purification step.

### NMR Spectroscopy

**Figure 4:**
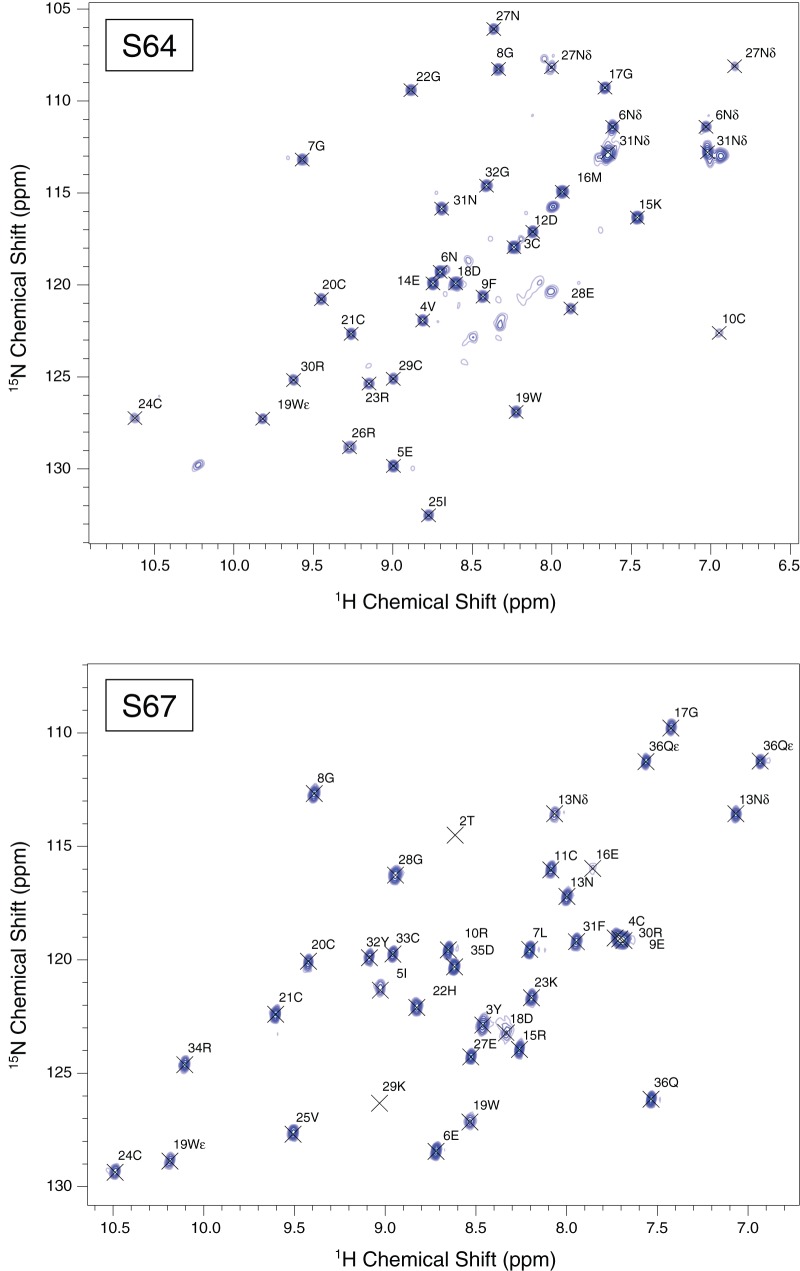
^15^N-HMQC spectra for S64 (**top**) **and S67** (**bottom**)**.** The S64 spectrum includes some minor peaks that are not assigned. These peaks do not have discernable cross-peaks in the NOESY spectra and only very weak cross-peaks in the 3D assignment spectra, which suggest that they are due to unfolded peptide rather than a minor conformation with a different disulfide-bonding pattern.

We assigned the ^15^N, ^13^C, and ^1^H resonances for the backbone atoms using band-selective excitation short transient [19], [20] (BEST) variants of the standard triple resonance sequences (HNCO, HN(CA)CO, HNCACB, HN(CO)CACB) and the ^13^C and ^1^H side-chain resonances using H(CCO)NH, CC(CO)NH, and HCCH-TOCSY spectra. We acquired two-dimensional NOESY as well as three-dimensional ^15^N NOESY-HSQC and ^13^C NOESY-HSQC experiments for generating distance constraints. We acquired most of the spectra for S64 and S67 at 600 MHz for ^1^H on Bruker NMR spectrometers equipped with conventional probes (for assignment spectra) or cryogenic probes (for NOESY spectra). A few two-dimensional spectra for S67 were acquired on a 750 MHz Bruker NMR spectrometer equipped with a cryogenic probe. We processed the spectroscopic data using TopSpin 2.1 (Bruker Biospin) and interpreted it using Analysis 2.2 [21] (Collaborative Computing Project for NMR). The sample temperature was maintained at 300 K (for S64) or 310 K (for S67) throughout the NMR experiments. Representative ^15^N HMQC spectra are shown in Figure 4.

For S67 we measured the rate of exchange for amide protons by dissolving a lyophilized ^13^C,^15^N-labeled sample in D_2_O and then immediately acquiring a series of ^15^N HMQC spectra. Amide protons that were slow to exchange (i.e., with exchange half-lives greater than approximately one hour) were assumed to be in strong hydrogen bonds.

### Structure Calculation

We used chemical-shift matched peak lists from the NOESY spectra along with TALOS+ derived torsion angle restraints [22] as input for ARIA 2.3 [23]. For later rounds of structure calculations, we included disulfide constraints as well as, for S67, hydrogen-bond constraints. We added these once the cysteine connectivity and identity of the hydrogen bond acceptors were obvious from previous iterations of the structure calculation. For the most part, the structure calculations followed the standard ARIA protocol, except that we used the log-harmonic potential [24], increased the number of cooling steps from 5000 to 15,000 for the first simulated annealing period (cool1) and from 4000 to 12,000 for the second (cool2), and calculated 30 structures (rather than 20) for each iteration. Statistics for the structural calculations are provided in Table 1.

**Table 1:** Structural statistics for the S64 and S67 ensembles.

			S64	S67
Physical Parameters (including nonnative N-terminal residue)				
	Number of residues		32	36
		Average molecular weight (reduced, unlabeled, Da)	3565.98	4249.82
		Monoisotopic molecular weight (reduced, unlabeled, Da)	3563.40	4246.85
Structural Restraints				
	NOE-derived distance restrains (ARIA cycle 8)			
		Intraresidue (|*i* – *j*| = 0)	209	319
		Sequential (|*i* – *j*| = 1)	66	94
		Short (2≤|*i* – *j*|≤3)	9	20
		Medium (4≤|*i* – *j*|≤5)	11	23
		Long (|*i* – *j*|>5)	34	83
		Ambiguous	84	137
		Total	413	676
	Dihedral constraints			
		Phi	24	25
		Psi	24	25
	S_γ_-S_γ_ distance restraints		3	3
	Hydrogen bond restraints		0	7
Statistics for accepted structures				
	Accepted structures		20	20
	Mean CNS energy terms			
		*E* total (kcal mol^–1^ ± SD)	−786 (±41)	−714 (±47)
		*E* van der Waals (kcal mol^–1^ ± SD)	−100 (±49)	−126 (±10)
		*E* distance restraints (kcal mol^–1^ ± SD)	183 (±15)	294 (±21)
	Restraint violations >0.3 Å (average # per structure)		5.6 (±1.7)	5.7 (±2.5)
	RMS deviations from the ideal geometry used within CNS			
		Bond lengths (Å)	0.0040	0.0043
		Bond angles (°)	0.55	0.64
		Improper angles (°)	1.86	3.00
Ramachandran Statistics (PROCHECK 3.5.4 [37])				
	Most favored (%)		88.5	68.6
	Additionally allowed (%)		10.8	25.9
	Generously allowed (%)		0.42	1.07
	Disallowed (%)		0.21	4.47
Average atomic RMS deviations from average structure (±SD)*				
	N, C_α_, C, and O atoms (all residues, Å)		0.86 (±0.22)	0.87 (±0.33)
	All heavy atoms (all residues, Å)		1.43 (±0.21)	1.33 (±0.16)
	N, C_α_, C, and O atoms (for residues with cop ≥0.9, Å)		0.57 (±0.17)	0.33 (±0.08)
	All heavy atoms (for residues with cop ≥0.9, Å)		1.30 (±0.17)	1.04 (±0.14)
MolProbity analyses (v3.19 [38])				
	Clashscore		12 (±4)	18.7 (±5.4)
	Clashscore percentile (%)		63 (±18)	39 (±19)
	Clashscore Z-score		0.42 (±0.62)	−0.25 (±0.48)

* Two sets of atomic RMS deviations are provided. The first set is for the full peptide (residues 1–32 for S64, 1–36 for S67) whereas the second set is calculated only including residues for which the circular order parameters (cop) for both φ and ψ are ≥0.9 (residues 2 and 4–30 for S64, and residues 5–35 for S67).

We deposited chemical shift assignments and restraints at the BioMagResBank (entry 18729 for S64 and 18600 for S67), and structure coordinates in the Worldwide Protein Data Bank (identification code 4B2V for S64 and 4B2U for S67).

## Results and Discussion

Using homology searching we identified peptides with characteristics consistent with known spider venom peptides in venom-expressed transcripts from *Sicarius dolichocephalus*. Although toxins have regions of high diversity, the cystine scaffolds that form the core of these peptides are largely conserved [25], [26]. Venom toxins often have fewer than 40 amino acids, so it is the cystine scaffold (rather than a hydrophobic core) that allows such small peptides to have a well-defined folded structure. Spider venom peptides are expressed as prepropeptides, with a signal sequence and a linker region that are cleaved after translation to produce the mature toxin. From the peptides we isolated that are candidate venom toxins we selected two homologous sequences, S64 and S67, for further study. These transcripts correspond to predicted mature peptides with six cysteines and average molecular weights (Da)/pI of 3,478.90/4.66 and 4,192.77/6.44 respectively. The sequences and the predicted cleavage sites for these peptides are shown in Figure 1.

Database searches had no hits with toxins or any other sequences in databases with expect values less than 10^−5^, a standard threshold for detecting homology (for example see [27]). These transcripts also did not hit any sequences in GenBank's nr or EST databases. However, when S64 and S67 are aligned with the closest matches in Arachnoserver, it is clear that they share the same cysteine motif and a conserved glycine (X_0–3_CX_3_GX_2_CX_4–9_CCX_2–7_CX_4–9_CX_1–3_) with toxins isolated from venom gland expressed transcripts in theraphosid and ctenid spiders. The theraphotoxins were identified when doing a search with S64, and had expect values between 0.14 (U6-theraphotoxin-Cj1a) and 0.67 (ω-theraphotoxin-Hh1a_1). The ctenitoxins are from a search with S67, and had expect values of 0.047 (U2-ctenitoxin-Co1a) and 0.39 (U23-ctenitoxin-Pn1a).

**Figure 5:**
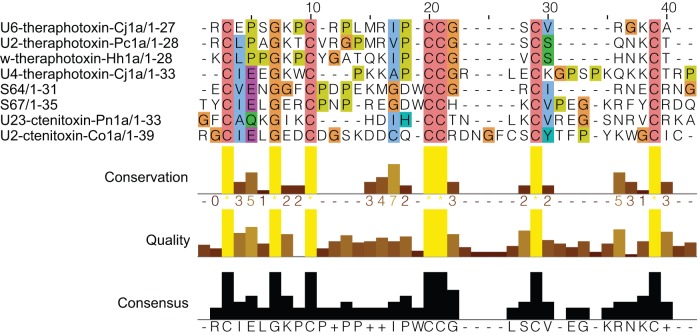
Sequence alignment for mature toxin sequences. This figure displays the mature toxin sequences for S64 and S67 aligned with representative toxins found using a BLASTp search using the Arachnoserver toxin peptide database. Sequences were aligned using ClustalX 2.1 and visualized using JalView 2.7. The coloring makes use of the default ClustalX color scheme, which is a function of sequence identity and amino acid type.

From the alignment (Figure 5), it can be clearly seen that aside from the cysteines and a few hydrophobic residues, there is little sequence similarity between S64/S67 and the other sequences. This is not necessarily surprising, considering that the divergence times between the species from which these peptides were identified exceed 200 million years [28]. In fact, the most recent common ancestor of these *Sicarius* peptides and theraphotoxins is the ancestor of all spiders except the basal lineage of Mesothele. However, S67 and, to a lesser extent S64, show strong sequence homology to other currently unpublished sequences that we have isolated from *S. dolichocephalus* as well as from other Haplogyne spiders (*Loxosceles spinulosa* and *Scytodes sp*., data not shown) indicating this toxin motif is broadly present among sicariids and close relatives.

**Figure 6:**
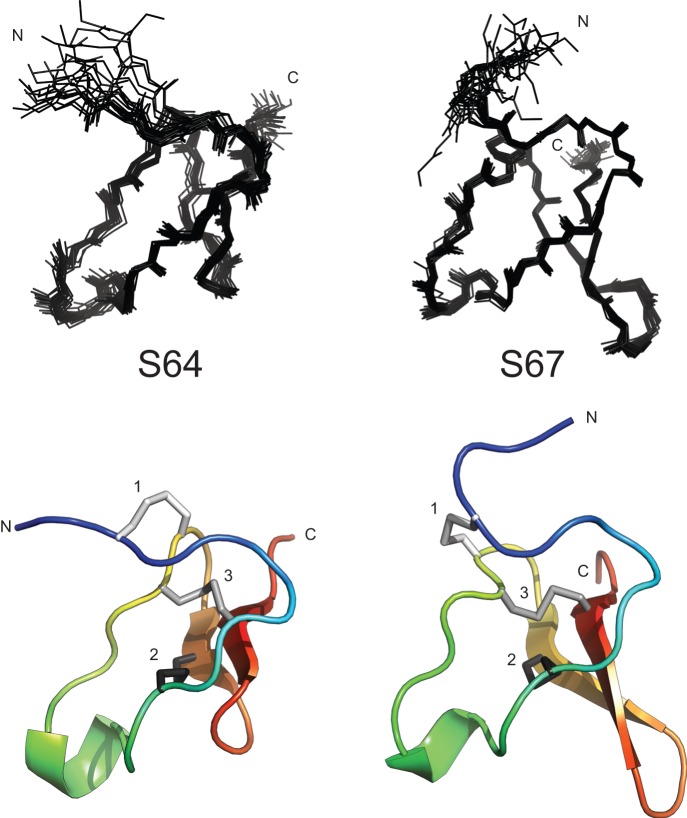
Ensemble (**top**) **and cartoon** (**bottom**) **representations of S64** (**left**) **and S67** (**right**)**.** The N and C termini are labeled in both the ensemble (top) and cartoon (bottom) representations of the structures of S64 (left) and S67 (right). The three disulfide bonds in the cartoon representations are also labeled. The ensembles represents the 20 structures with the lowest total energy out of 100 calculated structures. The cartoon representations show the lowest energy structure from each ensemble. Note that in the cartoon representation for S64 the third disulfide bond connects to the β sheet (red), not the strand (teal) in front of it.

We generated the structural ensembles shown at the top of Figure 6 by calculating 100 structures at the last stage of the ARIA protocol, performing a water refinement for each, and then selecting the 20 with the lowest total energy. The statistics for these ensembles, including the RMSD values, are given in Table 1. As can be seen in the cartoon representations at the bottom of Figure 6, the structures for both S64 and S67 follow the ICK peptide motif, with the six cysteine residues connected in a I–IV, II–V, III–VI pattern (cysteines numbered from N to C-terminus). This is not surprising, as many spider venom toxins as well as toxins from other organisms contain this structural motif. S67 follows the canonical ICK structure with standard loop sizes and is similar to ω-theraphotoxin-Hh1a [29], whereas S64 is somewhat more unique. Although S64 also follows the ICK motif, the β-hairpin is unusually truncated and, conversely, the second inter-cysteine region is longer than that found in most other venom toxins. This structure is somewhat reminiscent of ω-hexatoxin-Hv2a, a potent and specific insect calcium channel blocker [30], which also features a truncated β-hairpin loop. Despite the structural similarity, the sequences for S64 and a truncated version of ω-hexatoxin-Hv2a share only eight amino acids and have a BLASTp expect value of 10.

Although the structural ensembles for S64 and S67 clearly show the disulfide bonding topology, they do not show a clear trend in disulfide bond conformations (i.e., left-handed twist, right-handed spiral, etc.). This is not surprising for a spider venom toxin determined by NMR; analyses of the structures for huwentoxins [29] and many other spider toxins structures in the Protein Data Bank show similar results. This implies that the disulfide bonds are conformationally flexible and/or that the NMR restraints used to calculate the structures were insufficient for defining the disulfide bond conformations. There are some examples of spider toxin structures determined by NMR where the conformations of the disulfides are well defined between the structures in the ensemble [31]. In these cases, additional NMR experiments are typically used to measure proton-proton scalar couplings. The scalar coupling values can then be used to stereospecifically assign methylene protons and/or to restrict the dihedral angles available to sidechains in the structure calculation process. The resulting structural ensembles only show that the side chains have a preference for one conformation or another, and should not be interpreted as evidence of rigidity. To establish the conformational dynamics of the disulfide requires techniques such as NMR relaxation measurements; we plan to apply such measurements in the future to our toxins.

The peptides, as studied, differ slightly from the native mature peptides due to the engineered TEV cleavage site between the peptide and the rest of the fusion protein in the expression system. In the case of S64, this resulted in an additional N-terminal serine, whereas for S67 a N-terminal glycine was added. These additional residues, which are included in the statistics given in Table 1 and the structural models shown in Figure 6, slightly decrease the overall quality of the structure, as they are less well ordered than the native residues in the sequence. It is also possible that our estimate of the N-terminal cleavage site is inaccurate as it has not been experimentally confirmed. Many ICK spider venoms, especially the ones that align most closely with S64 and S67, have only a small number (0–2) of amino acids prior to the first cysteine of the mature toxin. Although for some spider venoms the site of cleavage can be predicted by the presence of a processing quadruplet motif (PQM) just before the cleavage site [26], the sequences from which the PQM were identified typically only have 1–3 amino acids before the first cysteine in the mature toxin. Consequently, we feel reasonably confident that we have identified the correct cleavage site for the mature toxin. However, even if we exclude a few amino acids that naturally occur at the N-terminus, their inclusion would likely have no affect on the overall structure of the peptide.

S64 and S67 are eukaryotic extracellular peptides with disulfide bonds that we recombinantly expressed intracellularly in a prokaryotic expression system, so it is possible that the disulfide bonds did not form correctly. The difficulty of achieving the correct fold for a venom toxin peptide increases with additional cysteine residues. For S64 and S67, which each have six cysteines, the number of possible folds are more limited compared to some of the other venom peptide targets that we work with, which have eight or even ten cysteines. In our case, S64 and S67 seem to adopt only a single folded confirmation, as indicated by a single well-resolved peak in the HPLC trace for these peptides (Figure 2), and the presence of a single set of peaks in the NMR spectra (Figure 4). In addition, we confirmed that the cysteines were oxidized, and therefore in disulfide bonds, using mass spectroscopy. In the mass spectra (Figure 3), the masses were six atomic mass units lower than expected if the peptides were completely reduced. This mass difference indicated that all six cysteines in each peptide were oxidized. Finally, the chemical shifts from our NMR spectra for the cysteine α and β carbons were consistent with oxidized, rather than reduced, cysteines [32]. As spider venom toxins have been shown to adopt the correct fold when folded *in vitro* [33], [34], [35], we believe that we have produced a single, fully oxidized product for both S64 and S67 and that the peptides have adopted their native fold.

## Conclusion

We have presented the first structures for putative venom peptides from *Sicarius* spiders. These peptides, which were identified by screening cDNA libraries made from venom gland mRNA, exhibit structures that follow the ICK motif that is common to many venom peptides from spiders and other organisms. The broad conservation of the six-cysteine motif shown in Figure 2 across a set of species whose most recent common ancestor includes the ancestor of all spiders (except the basal lineage of mesotheles) is consistent with a single evolutionary origin and conservation of this specific motif. While with this taxon sampling we cannot definitively rule out independent convergence, the conservation across three highly divergent lineages is more likely than three separate origins. Although the functions of our peptides are currently unknown, based on homology they are likely to be neurotoxic and to target ion channel proteins. In the future, we plan to functionally characterize the peptides using electrophysiological and fluorescence assays.
